# The complexity of opportunities to respond used by mothers and fathers of children with Down syndrome: A preliminary investigation

**DOI:** 10.1017/S0305000924000370

**Published:** 2024-09-27

**Authors:** Marianne Elmquist, Andrea L.B. Ford, Audra Sterling

**Affiliations:** 1 Waisman Center, University of Wisconsin-Madison, Madison, WI; 2 Communication Sciences and Disorders, University of Cincinnati; 3 School of Communication Sciences and Disorders, University of Wisconsin-Madison, Madison, WI

**Keywords:** caregiver-child interactions, opportunities to respond, Down syndrome

## Abstract

Caregiver-child interactions are commonly used to examine children’s language learning environment. However, few studies consider interaction configurations beyond dyadic interactions or explore the conceptual complexity of caregiver talk. Thus, we examined if the complexity of a caregiver’s opportunities to respond (OTR) varied when sampled across three interaction configurations. Our study included twelve preschool-aged children with Down syndrome and both of their biological parents. Our preliminary findings suggest no differences in mothers’ and fathers’ frequency of OTRs across complexity levels during dyadic interactions. However, caregivers produced fewer OTRs across complexity levels during family choice than dyadic interactions.

## Introduction

Down syndrome (DS) is the most common chromosomal cause of intellectual disability, with a prevalence of one in 691 live births (Parker et al., 2010). In addition to intellectual impairments, co-occurring language delays and impairments impact multiple aspects of language comprehension and production (Abbeduto et al., 2007; Roberts et al., 2007). Moreover, these impairments present early in life for children with DS and persist throughout the lifespan. Given these language delays and impairments, early caregiver-child interactions are critical in providing the foundations for language learning for children with DS (Smith et al., 2020; Woynaroski et al., 2014).

Grounded in the transactional model of development (Sameroff, 2010), there is a wealth of research in support of the substantial and cumulative impact of caregiver-child interactions on child language development both for neurotypical (NT) children and children with disabilities in Western countries (Bottema-Beutel et al., 2021; Bozicevic et al., 2023; Ford et al., 2020; Pace et al., 2022; Woynaroski et al., 2014). During these interactions, caregivers use various discrete language-facilitating strategies for different purposes that promote language learning and provide opportunities for children to demonstrate their communicative skills. These strategies include but are not limited to, expansions, follow-in comments, linguistic mapping, mirroring, modeling, and opportunities to respond (Elmquist et al., 2024; Kaiser & Roberts, 2013; Mattie & Hadley, 2021; McDuffie & Yoder, 2010). Given the importance of caregiver-child interactions, researchers and clinicians often examine (and intervene at) the structural features of caregiver talk (e.g., lexical diversity and complexity) to optimize language learning. Towards that premise, Rowe and Snow (2019) discussed how caregiver talk can be analyzed along three dimensions that provide a framework for the current study. These dimensions are: (a) conceptual, such as conversational topics that provide a developmentally appropriate challenge (e.g., concrete/contextualized versus abstract/decontextualized), (b) interactional, such as features that support the child’s involvement (e.g., responsiveness, shared attention), and (c) linguistic or the phonological, lexical, and grammatical features of caregiver talk (Rowe & Snow, 2019). In this investigation, we narrowed our focus to two dimensions: 1) conceptual, 2) interactional. Specifically, we focused on the conceptual complexity of opportunities to respond (OTRS; Greenwood et al., 1984). OTRs can be defined as caregiver communicative bids (e.g., questions, comments) that create openings for children to interact with their communication partners and, importantly, provide opportunities to demonstrate their communication and language skills (Ford et al., 2020). We opted to focus on explicit OTRs (i.e., questions) given that asking questions compared to comments increase children’s response rates (Yoder & Davies, 1990; Yoder et al., 1994). Children’s responses, in turn, provide additional opportunities for caregivers to continue the interaction.

Research examining the conceptual features of caregiver talk – and how it relates to child outcomes – among children with DS is limited. Most recently, Hilvert et al. (2021) found that mothers of children with DS tended to use the same proportion of decontextualized talk as mothers of NT children across three routines of free play, shared book reading, and snack, with the exception of pretend talk (e.g., the baby is hungry). They further found that mothers’ decontextualized talk was not associated with children’s chronological or developmental language ability. These same researchers also examined mothers’ and fathers’ decontextualized and contextualized talk during shared book reading among children with DS (Hilvert et al., 2022). Their results showed no differences in the type of talk between caregivers, except the fathers produced more reading utterances compared to mothers. Notably, they found that children’s expressive language and lexical diversity were associated with fathers’ use of decontextualized talk, not mothers. As a robust and vital facilitator of language learning, we sought to examine the extent to which caregiver OTRs were grounded in the here and now (i.e., contextualized talk) or involved more abstract and often more complex concepts that were not grounded in the here and now (i.e., decontextualized talk; Snow, 1991) in caregiver-child interactions.

## Interaction configuration and caregiver talk

Within the caregiver-child interaction literature, researchers have primarily measured dyadic interactions, with a significant portion focused solely on mother-child dyads (e.g., Hilvert et al., 2021; Peredo et al., 2020). However, many children are likely to interact with multiple caregivers and siblings. Families composed of mother-father, two-caregiver households are just one example. Understanding how these interactions may change depending on the caregiver is critical, given that an emerging body of literature describes differences and similarities in mothers’ and fathers’ talk (e.g., Hilvert et al., 2022; Pancsofar & Vernon-Feagans, 2006). For example, mothers of children with DS provided more language and descriptive language than fathers during dyadic free play sessions (de Falco et al., 2011). These findings also extend to shared book reading: fathers spend more time reading the book text than mothers, who are more talkative and use more descriptive language (Hilvert et al., 2022). Furthermore, maternal and paternal language input was associated with different aspects of child language during book reading, suggesting that caregivers support language acquisition in divergent approaches (Hilvert et al., 2021).

Different interaction configurations (e.g., dyadic versus triadic interactions) are also associated with differences in the nature and frequency of caregiver talk. For example, research including NT children has shown that during dyadic interactions, mothers’ and fathers’ words and utterances are more complex than their talk in triadic interactions (Nandy et al., 2021; Quigley & Nixon, 2022). Elmquist et al. (2024) found that when combining mothers’ and fathers’ communicative bids, children with DS were exposed to fewer bids during interactions involving mothers, fathers, and siblings (if present) compared to dyadic caregiver-child interactions. Furthermore, there were differences in this effect based on the explicitness of the communicative bid (i.e., questions versus comments).

### Caregiver-child interaction data collection methods

There are two general approaches to collecting caregiver-child interactions. The first is in-person observations that often involve an examiner being present and are typically collected at home or a research lab (e.g., Bottema-Beutel et al., 2018; Potter et al., 2024). Standardized materials (e.g., toys, books) are often provided and caregivers are instructed to interact with their child as they typically would without an examiner present (e.g., Bottema-Beutel et al., 2018). This approach often captures dyadic interactions, with mother-child dyads being the most common configuration studied. The second method involves long-form recordings without an examiner present (Casillas et al., 2019). These long-form recordings often utilize LENA recordings (e.g., Gilkerson et al., 2017; Parikh & Mastergeorge, 2018) and caregivers are typically instructed to go about their typical routines but to remain at home (e.g., Thiemann-Bourque et al., 2014). With these long-form recordings, there is opportunity to examine a variety of interaction configurations, such as mother-child, father-child, sibling-child, or even multiple caregivers/siblings and the focal child.

A comprehensive picture of the child’s communication interactions is a clear benefit of long-form recordings. Caregiver-implemented communication interventions are often taught and examined in dyadic configurations, but they are expected to generalize to interactions beyond these dyadic interactions. If we want to actualize this generalization instead of training and hoping (Stokes & Baer, 1977), we must examine caregivers’ and children’s communication in interactions that represent the configurations in which they will be implemented at the outset. In doing so, we can better understand language learning opportunities for children with DS and improve the generalized efficacy of future caregiver-implemented communication interventions.

### Study purpose and research questions

To further our understanding of the language environments of children with DS, we must examine dimensions of language-facilitating strategies, such as the conceptual complexity of caregiver talk. However, to ensure we are comprehensive in our measurement, it is equally critical that we examine how language dimensions might change across different interaction configurations. This preliminary investigation examined if there were differences in the conceptual complexity of mothers’ and fathers’ use of one strategy – OTRs – across interaction configurations (e.g., dyadic free play video observations vs. family choice LENA observations). The current research questions (RQ) guided the present study:

- RQ1: Are there differences in the conceptual complexity of OTRs between mothers and fathers during dyadic caregiver-child interactions?
- RQ2: Do mothers’ and fathers’ produce fewer conceptually complex OTRs in family choice interactions compared to dyadic caregiver-child interactions?
- RQ3: Are there differences in the conceptual complexity of OTRs children are exposed to during dyadic versus family choice interactions when mothers’ and fathers’ OTRs are combined?

## Methods

### Participants

Twelve children with DS (*M* = 3 years; 4 months, *SD* = 12.16 months) and their biological parents participated in the current study. This age range was selected because during the preschool years children’s language becomes more complex and parent’s use of decontextualized talk increases (e.g., Rowe, 2012). Participants for the present study came from an existing dataset (Elmquist et al., 2024) and included participants from a larger, previously published dataset (*n* = 15; see Hilvert et al., 2022; Lorang et al., 2020; Lorang & Sterling, 2021). We included families in the current study if they had completed the following caregiver-child interactions: dyadic mother-child freeplay, dyadic father-child freeplay, and family choice LENA interactions. Caregivers provided genetic documentation of trisomy 21. Children lived in two-caregiver households and had normal or corrected hearing and vision. All families were white, non-Hispanic/Latino, and monolingual English-speaking. Families were recruited from clinics, centers, and early intervention providers serving families with DS in Madison, Wisconsin, USA, and the surrounding areas. We used the Receptive and Expressive subscales of the Mullen Scales of Early Learning (MSEL; Mullen, 1995), to characterize children’s language abilities. A trained examiner administered the MSEL during one of the two home visits. Table 1 provides complete demographic information.

### Procedures

The University’s Institutional Review Board approved the study, and written informed consent was obtained from both parents. All examiner-led study procedures were completed in the family’s home over two visits.

#### Caregiver-child interactions

We employed two commonly used methods to collect caregiver-child interactions for the current study: video observations collected in the home for dyadic caregiver-child interactions (e.g., Hilvert et al., 2022; Leezenbaum et al., 2014) and a LENA recording for the family choice context (e.g., Parikh & Mastergeorge, 2018; Thiemann-Bourque et al., 2014). Ten-minute dyadic interactions were video-recorded separately for mother-child and father-child interactions during free-play observations with an examiner present. Families were provided with a developmentally appropriate standardized toy set, and caregivers were instructed to “play as you typically would” but to only play with the included items (see supplemental material for a list of toys used). The order of dyadic interactions was randomized across participants. We used LENA recorders to collect family choice interactions without an examiner present. This method was used to capture more naturalistic family interactions. Families completed a 3-hour LENA recording during an evening, and this was completed before the first examiner visit. Each family was given instructions, a LENA recorder, and specialized clothing to hold the LENA. Caregivers were instructed to go about their everyday family routines (e.g., play, meal-times, laundry). Both caregivers were present; in five instances, siblings were also present.

#### Transcription and coding

Trained research assistants from a communication disorders program completed all transcriptions and coding. Transcribers and coders completed a standardized training protocol using caregiver-child interactions not included in the current dataset. There was some overlap between coders and transcribers, but all were blinded to the study research questions. Coders started independent work after completing three consecutive training transcripts at or above 80% reliability across all OTR and transcription measures.

All interactions were transcribed using the Systematic Analysis of Language Transcripts (SALT) conventions and software (Miller et al., 2011). While LENA recordings provide automated measures of adult and child vocal behaviors, we transcribed a portion of the recording, adapting procedures from Wood et al. (2016). Specifically, a bar graph of vocalizations of the 3-hour recording was visually inspected to identify a high-volubility 10-minute segment (i.e., a segment containing the greatest amount of caregiver and child talk). This segment was then transcribed using SALT conventions. Some dyadic recordings were shorter than the planned 10-minutes; to ensure consistency across participants and conditions, we only used the first 8-minutes (the longest time length for one participant) of all interactions for analyses.

#### Coded variables

In this study, we defined OTRs as caregiver utterances that provided an explicit response pathway for a child (i.e., questions). These had been previously coded in Elmquist et al. (2024). For the current project, we coded each OTR for its conceptual complexity using codes adapted from Massey et al. (2008). We had three mutually exclusive codes: management, less conceptually challenging (LLC), and more conceptually challenging (MCC). Of note, OTRs that were coded as MCC are also likely to include more complex syntax compared to management or LCC OTRs. See Table 2 for operational definitions that include examples of what was coded for each code and what was not. Our full coding manual is included in supplemental materials.

#### Reliability

We obtained reliability data for the transcription and coding process. For transcriptions, 24% of interactions were transcribed by a second transcriber. Line-by-line percent agreement was calculated for segmentation, number of morphemes, number of words, word identification, and intelligibility (*M* = 88.75%, min-max: 85.92–94.48%). For OTR complexity coding, 28% of interactions were initially coded by a second coder. Reliability was below 80% (*M* = 57%, min-max: 8–92%) due to the low base rates of the MCC code. As a result, all explicit OTRs were consensus coded for complexity codes.

### Data analysis

Given our small sample size, multiple comparisons, and dependencies between comparisons, we calculated Hedges *g* and 95% confidence intervals (CIs) to examine differences in caregiver OTRs across interaction configurations (Nakagawa & Cuthill, 2007; Patriota, 2017). We interpreted effect sizes as small (*g* = .20), medium (*g* = .50), and large (*g* = .80; Cohen, 1988). Specifically, we compared differences in mothers’ and fathers’ complexity of OTRs between mother-child and father-child dyadic interactions and within caregiver differences across dyadic and family choice interactions (e.g., mother dyadic versus mother family choice). For our third research question, we were interested in how interaction configurations impacted the complexity of OTRs directed to the child. Therefore, we compared the OTRs produced by mothers and fathers in their respective dyadic interactions with the combined OTRs of mothers and fathers in family choice interactions.

## Results

**Figure 1.**
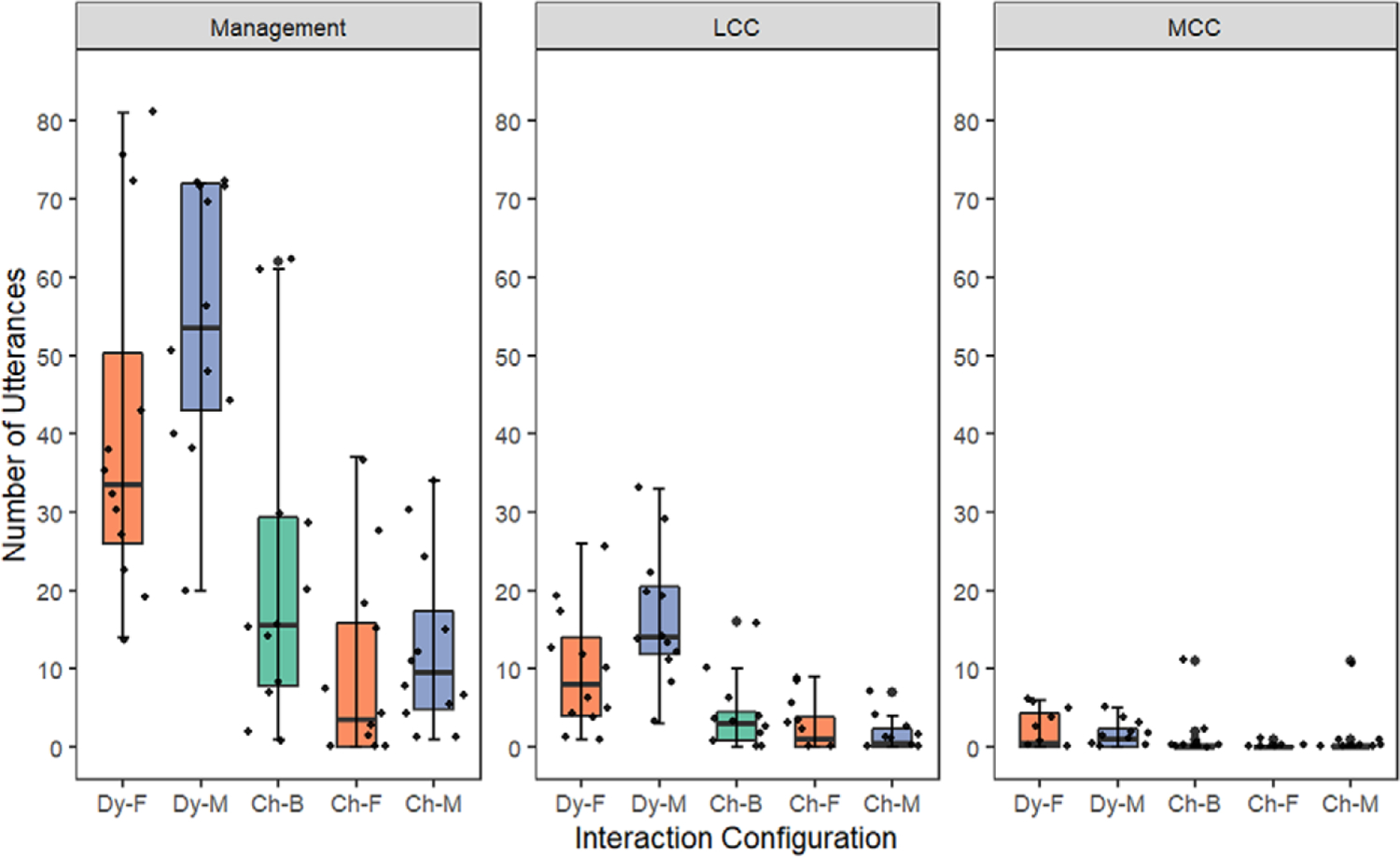
Number of OTRs Across Complexity Levels and Interaction Configuration *Note*. Dy-F = father OTRs during dyadic father-child interaction. Dy-M = mother OTRs during dyadic mother-child interaction. CH-B = mother and father OTRs combined during family choice interaction. Ch-F = father OTRs during family choice interaction. Ch-M = mother OTRs during family choice interaction. LLC = less conceptually challenging. MCC = more conceptually challenging.

Table 3 displays means, standard deviations, and ranges for the conceptual complexity of caregivers’ OTRs across interaction configurations. Across caregivers and interaction configurations, more OTRs were classified as management (mean range: 12.46–54.58), followed by less conceptually challenging (mean range: 1.5–16.50-) and more conceptually challenging (mean range: 0.08–2.08-). Given the large observed standard deviations across study measures – suggesting inter-caregiver variability – we also examined individual caregiver data to understand how caregivers may have contributed to group results (see Figure 1). Table 4 displays the results for Hedges *g* and 95% confidence intervals.

### RQ1: Mother versus father dyadic comparisons

We observed medium effects of fathers producing fewer management OTRs than mothers during dyadic caregiver-child interactions. This finding was the same for less conceptually challenging OTRs. In contrast, we observed a small effect for fathers producing more conceptually challenging OTRs than mothers. However, confidence intervals contained zero for all results, indicating that these effects were not statistically significant had we calculated *p*-values.

### RQ2: Within caregiver differences between dyadic and family choice interactions

Mothers and fathers produced fewer OTRs across complexity classification during family choice interactions than their respective dyadic caregiver-child interactions. We observed large effects for management and less conceptually challenging OTRs. For more conceptually challenging OTRs, we observed large effects for fathers and negligible effects for mothers. For all analyses, except for the mother’s more conceptually challenging OTRs, confidence intervals did not contain zero, indicating differences are likely to be meaningful.

### RQ3: Combined caregiver OTRs in family choice compared to dyadic interactions

We observed large effects of children being exposed to fewer management OTRs when combining mothers’ and fathers’ management OTRs compared to their respective dyadic interactions. Similarly, we observed large effects for less conceptually challenging OTRs. Small effects were observed when comparing combined family choice with the father’s dyadic use of more conceptually challenging OTRs and negligible effects were observed for mothers. For all results but the mother’s use of management and less conceptually challenging OTRs, confidence intervals contained zero, suggesting differences observed are not meaningful.

## Discussion

Caregiver-child interactions are essential, robust, and facilitative contexts for promoting language learning in young children with DS. Furthermore, research suggests that mothers and fathers use different approaches to support language development (e.g., de Falco et al., 2011; Hilvert et al., 2022; Pancsofar & Vernon-Feagans, 2006). However, a paucity of studies have examined the conceptual complexity of mothers’ and fathers’ utterances across interaction configurations to which young children with DS are exposed. We aimed to understand if there were conceptual complexity differences in mothers’ and fathers’ OTRs across interaction configurations.

For our first research question, we compared mothers’ and fathers’ OTR complexity during dyadic caregiver-child interactions. Effect sizes indicated that mothers produced more management and less conceptually challenging OTRs but fewer more conceptually challenging OTRs than fathers. These effects, however, are unlikely to be meaningful given our confidence intervals. As such, we have preliminary evidence that there may be no differences among mothers’ and fathers’ use of a range of conceptually complex OTRs during dyadic free-play interactions. These results align with the findings of Hilvert et al. (2022), who similarly found no differences amongst caregivers across most types of talk during shared book reading – however, there was overlap between the two samples. While more exploration and replication is needed, we have initial evidence that caregivers’ style of talk may be consistent across dyadic interaction contexts. If this finding is replicated, ideally with larger more diverse samples, it will likely have implications for intervention and generalization.

We found that while fathers produced a higher frequency of more conceptually challenging OTRs compared to mothers during dyadic interactions, these results are unlikely to be meaningful in our study. This finding adds to the conflicting evidence in the literature: some studies showing differences in fathers and mothers decontextualized talk (i.e., talk not grounded in the here and now; e.g., Duursma, 2016) while others show no difference (e.g., Hilvert et al., 2022). More research is needed to explore if meaningful differences exist between mothers and fathers in the conceptual complexity of their talk, and if so the implication of those differences.

The present study also examined caregiver differences across interaction configurations (i.e., RQ2). We found large and meaningful effects for mothers and fathers producing fewer management and less conceptually challenging OTRs during family choice interactions than their respective dyadic interactions. We also found significant and meaningful effects for fathers producing fewer more conceptually challenging OTRs during family choice interactions than father-child interactions. In contrast, negligible effects were observed for mothers. This is unsurprising given that mothers did not use many of the more conceptually challenging OTRs regardless of interaction configuration. Our findings from our second research question align with the limited NT research examining dyadic and triadic caregiver-child interactions. Nandy et al. (2021) also found that parents produced fewer utterances during triadic interactions than their dyadic interactions. We would also expect interactions involving more communication partners (e.g., multiple caregivers and siblings) to result in fewer opportunities for one-on-one caregiver-child interactions (McHale & Fivaz-Depeursinge, 1999).

Lastly, we examined if children with DS were exposed to differing amounts of OTRs across complexity levels during dyadic and family choice interactions (i.e., RQ3). We found that children were exposed to fewer OTRs across complexity levels during more naturalistic family contexts (i.e., family choice), compared to dyadic interactions, even when combining caregiver OTRs. However, our results suggest that these differences are not meaningful for most comparisons, except for mothers’ use of management and less conceptually challenging OTRs. These findings diverge from Nandy et al. (2021), who found that children during triadic interactions were exposed to more caregiver utterances when combining mothers’ and fathers’ utterances than dyadic interactions. This difference might be due to child characteristics (e.g., DS versus NT), different approaches to measuring caregiver talk, the context of interactions, or because some of our family choice interactions also included siblings (*n* = 5).

Overall, our findings present preliminary evidence that interaction configuration has a greater impact on differences in caregiver talk than communication partner (i.e., mother versus father). These findings have important implications for caregiver-mediated communication interventions. These interventions are often taught and examined in dyadic contexts yet are intended to be implemented in contexts that extend dyadic interactions. Our results suggest that the dosage of these interventions may be influenced by interaction configuration. More research is needed to understand how interaction configurations influence child language learning among children with DS and the impact on intervention implementation and efficacy.

### Limitations and future research

The present study had several limitations, which should be considered when interpreting the results. First, the small sample size included children with a relatively wide age range and considerable caregiver talk variability. As a result, we had large CIs, which impacted the precision of our effect sizes and reduced our findings’ generalizability. This is particularly important given the known heterogeneity in language profiles observed among children with DS. Second, our study comprised white, mono-English, heterosexual caregivers. To increase the generalizability of future research, research that is more inclusive of individuals from marginalized backgrounds is needed. Third, we cannot determine if the recording mode of caregiver-child interactions influenced differences in our results. Future research must examine whether the method used to collect caregiver-child interactions influences our measurement of caregiver and child talk. Lastly, we need longitudinal research across interaction configurations to test and identify caregiver talk associated with language learning that can be leveraged in future caregiver-mediated communication interventions.

## Conclusions

Our preliminary evidence suggests that mothers and fathers did not differ in using OTRs across complexity levels during dyadic caregiver-child interactions. In contrast, interaction configuration did influence caregivers’ frequency of OTRs across complexity levels, with caregivers producing fewer OTRs during family choice interactions compared to dyadic interactions. While children with DS are likely to be exposed to fewer OTRs across complexity levels during family choice interactions, these differences may not be meaningful depending on the type of OTR. While these findings need to be replicated, our results suggest that interaction configurations need to be carefully considered in the development of caregiver implemented communication interventions. Additionally, more descriptive research – concurrent and longitudinal – is needed to gather a holistic understanding of how interaction configurations impact aspects of caregiver talk beyond conceptual complexity.

## Supplementary Material

Supplementary Material 1

Supplementary Material 2

Supplementary Material 3

## Figures and Tables

**Table 1.** Participant Characteristics (n = 12)

	N (%) Unless other noted
**Child Characteristics**	
**Sex – female**	6 (50%)
**Mullen Scales of Early Learning**	
Receptive Language raw score – M (SD)	22.5 (6.76)
Receptive Language t–score – M (SD)	26.42 (8.98)
Expressive Language raw score – M (SD)	16.75 (5.91)
Expressive Language t–score – M (SD)	21.08 (2.35)
Cognitive Scale t–score – M (SD)	91.42 (17.11)
**SALT Language Measures** ^a^	
Mean length of utterance – morphemes – M (SD)	1.08 (0.45)
Number of total words – M (SD)	70.92 (66.93)
Number of different words – M (SD)	14.33 (19.23)
**Siblings**	
Target child with no siblings	3 (25%)
Mean (SD) number of siblings; range	1.33 (1.37); 1–5
**Caregiver Characteristics**	
**Age in years (SD)**	
Mother	40 (4.64)
Father	40 (5.85)
**Employment**	
Mother: Full–time	7 (58)
Mother: Part–time	2 (16)
Mother: Stay at home	3 (25)
Father: Full–time	9 (75)
Father: Part–time	1 (8)
Father: Stay at home	2 (16)
**Household Income**	
$25, 000 – $50, 000	2 (16)
$50, 000 – $75, 000	2 (16)
$75, 000 – $100, 000	2 (16)
$100, 000 – $150, 000	3 (25)
$150, 000 – $250, 000	2 (16)
More than $250, 000	1 (8)

*Note*. Mullen Scales of Early Learning (MSEL; Mullen, 1995). SALT = Systematic Analysis of Language Transcripts (Miller et al., 2011).

a Derived from dyadic father-child interaction.

**Table 2.** Definitions and Examples for Coding Conceptual Complexity of Caregiver OTRs

Code	Definition	Examples/Non-Examples
Management (M)	Questions that maintain conversation, manage behavior, clarify, and provide directives.	**Examples:** Ready to start reading our book?; What else? Say ‘ball’ **Non-Examples:** What is that? What color is the train
Less conceptually challenging (LCC)	Questions about information that is perceptually available or that offers concrete choices.	**Examples:** Do you want juice or water?; What color is the bus?; What is that?; What’s wrong? **Non-examples:** What else?; What happened next?
More conceptually challenging (MCC)	Questions about non-present objects or past and future events. Questions require the child to draw inferences, analyze information, discuss vocabulary, or make predictions.	**Examples:** How do you think the child is feeling?; What else did you do at the zoo? **Non-example:** What else?; And then what?

**Table 3.** Descriptive Statistics for Caregiver OTR Complexity

	Mother		Father		Combined
	Dyadic	Family Choice	Dyadic	Family Choice	Family Choice
	M (SD) Range	M (SD) Range	M (SD) Range	M (SD) Range	M (SD) Range
Management	54.58 (17.33)	12.67 (11.09)	30.83 (22.90)	9.42 (12.48)	22.08 (20.56)
	20–72	1–34	14–81	0–37	1–62
LCC	16.50 (8.57)	1.5 (2.20)	9.83 (7.83)	2.58 (3.34)	4.08 (4.74)
	3–33	0–7	1–26	0–9	0–16
MCC	1.5 (1.73)	1.08 (3.15)	2.08 (2.54)	0.08 (0.29)	1.17 (3.16)
	0–5	0–11	0–6	0–1	0–11

*Note*. LCC = Less conceptually challenging. MCC = more conceptually challenging

**Table 4.** Hedges g and 95% Confidence Intervals for Caregiver OTR Complexity

	Mother vs. Father Dyadic	Mother dyadic vs. mother family choice	Father dyadic vs. father family choice	Mother Dyadic vs. Combined Family Choice	Father dyadic vs. combined family choice
	ES [CI]	ES [CI]	ES [CI]	ES [CI]	ES [CI]
Management	−0.65	2.78	1.64	−1.65	−0.83
	[−1.49, 0.19]	[1.64, 3.93]	[0.70, 2.59]	[−2.60, −0.70]	[−1.68, 0.02]
LCC	−0.78	2.32	1.16	−1.73	−0.86
	[−1.63, 0.06]	[1.26, 3.37]	[0.28, 2.05]	[−2.69, −0.77]	[−1.71, 0.00]
MCC	0.26	0.16	1.07	−0.13	−0.31
	[−0.56, 1.08]	[−0.66, 0.98]	[0.19, 1.94]	[−0.94, 0.69]	[−1.13, 0.51]

*Note*. LCC = Less conceptually challenging. MCC = more conceptually challenging.
