# Supplementary material for: The complexity of opportunities to respond used by mothers and fathers of children with Down syndrome: A preliminary investigation
Source: J Child Lang. Author manuscript; Available in PMC 2025 Aug 2. (PMC11946924; doi:10.1017/S0305000924000370)
Supplement: Supplementary Material 1 [file NIHMS2041977-supplement-Supplementary_Material_1.docx]

**List of Toys available During Dyadic Caregiver-Child Interactions**

- Dinosaur set
- Wooden puzzle
- Teapot set
- Play food
- Baby doll
- 2x24 piece box of blocks
- Farm set
