# Supplementary material for: The complexity of opportunities to respond used by mothers and fathers of children with Down syndrome: A preliminary investigation
Source: J Child Lang. Author manuscript; Available in PMC 2025 Aug 2. (PMC11946924; doi:10.1017/S0305000924000370)
Supplement: Supplementary Material 2 [file NIHMS2041977-supplement-Supplementary_Material_2.docx]

**Opportunities to Respond Coding Manual**

This document contains the coding definitions, coding process and some tips and tricks for coding opportunities to respond based off SALT transcript files. Behavioral coding will be completed within an excel copy of the transcript. The following codes only apply to utterances spoken by anyone other than the target child, denoted with ‘C’ in the transcripts.

**Definitions**

**Child Directed Speech (CDS) vs Other-directed speech (ODS) vs Unclear (UN)**

- Child-directed speech (CDS) are utterances spoken by a communicative partner with intent of initiating or maintaining an interaction with the target child.
- Other-directed speech (ODS) are utterances spoken by a communicative partner during an interaction that are directed towards a communicative partner other than the target child (e.g., other family member) and they are not intended to evoke a response from the target child.
- In cases where it its is unclear if the utterance is child-directed or other-directed the utterance can be marked as unclear (UN). These should be used sparingly. Utterances that are entirely unintelligible will always be marked as unclear.

**Opportunities to Respond (OTRs)**

OTRs can be defined as *explicit* and *implicit* adult utterances that serve as prompts to evoke child *expressive* and *receptive* language behaviors.

**Expressive (EX)**

- **Explicit OTRs:** include questions or comments that provide direction on how and what the child response should look like (e.g., Do you want to read this book?).
- **Implicit OTRs**: are statements that directed to the focal child, but do not provide clear guidance on how or if the child should respond (“It is cold out today”).

**Receptive (RE)**

- Includes statements that are directive in nature such as requests for behavioral complies. (e.g., “pet the cat”, “look at this”). These statements provide information on how the child should respond, but do not require an expressive response.
  - Note that receptive OTRs are not broken down any further.

**Type of Expressive OTRs**

The following expressive OTR codes have been adapted from Ford, A. L., & Johnson, L. D. (2021). The use of generalizability theory to inform sampling of educator language used with preschoolers with Autism Spectrum Disorder. Journal of Speech, Language, and Hearing Research, 64(5), 1748-1757.

| *Definition and examples for coding the type of caregiver Expressive OTRs* | | |
| --- | --- | --- |
| Code (acronym) | Definition | Examples/Non-Examples |
| **Explicit (OTR-E)** | | |
| Open-Ended Questions (OEQ) | A question used by the caregiver that is directed at the focal child and the child’s answers is not restricted by length or content of the response. | **Examples**  What book should we read?  Tell me about your day at preschool?  Where should we go this afternoon?  What sound does a cat make?  What is that?  **Non-Examples**  Can you say ‘ball’?  Do you want to go outside? |
| Yes/No Questions (YNQ) | A question used by the caregiver that is direct at the focal child and is designed to be answered with a yes or no. Can be formal or informal. | **Examples**  Do you like this toy?  It is cold outside, isn’t it?  Do you want to read a book?  **Non-Examples**  What is your favorite animal?  Pet the cat |
| Choice (CH) | A question used by the caregiver that is directed at the focal child that offers at least two explicit options that the child may choose from using verbal responses. | **Examples**  Do you want to play with blocks or trains?  Do you want to read a book, play outside or color?  Do you want juice or water?  **Non-Examples**  Do you want juice?  What should we do tomorrow? |
| Imitation (IM) | A comment or question used by the caregiver that explicitly requests the child to repeat the word of phrase. | **Examples**  Say “ball”  Can you say “bye Teddy”  **Non-Examples**  Back and forth imitations between two speakers e.g.  F: meow  C: meow  F: meow |
| **Implicit (OTR-I)** | | |
| Comment (C) | A statement used by the caregiver that is directed at the focal child that carries meaning in its use, (e.g., it may describe something or label an object), but there would be no explicit communication breakdown if the child did not respond. | **Examples**  It is snowing outside.  That is a long train track.  The dog says Woof Woof.  **Non-Examples**  Bye  Rwaar  Awww |
| Other (O) | These are single words that do not fit in the categories above and may include exclamations, affirmations, greetings, or sound effects. | **Examples**  Oh-Oh!  Beep Beep.  Meow  **Non-Examples**  I like that picture  That is my favorite book |

**Complexity of Expressive Explicit OTRs**

Complexity of expressive OTRs codes have been adapted from Massey, S. L., Pence, K. L., Justice, L. M., & Bowles, R. P. (2008). Educators' use of cognitively challenging questions in economically disadvantaged preschool classroom contexts. Early Education and Development, 19(2), 340-360.

| *Definitions and examples for coding conceptual/cognitive complexity of caregiver expressive OTRs* | | |
| --- | --- | --- |
| Code | Definition | Examples/Non-Examples |
| Management (M) | Questions that maintain conversation, manage behavior, clarify, and provide directives | **Examples**  Can you pet the dog?  Ready to start reading our book?  What else?  And then what?  What did you say?  Say ‘ball’  **Non-Examples**  What is that? |
| Less cognitively challenging (LCC) | Questions about information that is perceptually available, or that offer concrete choices | **Examples**  Do you want juice or water?  What color is the train?  What is the child doing?  What is that?  What’s wrong?  **Non examples**  What else?  What happened next? |
| More cognitively challenging (MCC) | Questions about nonpresent objects or past and future events. Questions require the child to draw an inference, analyze information, discuss vocabulary, or make predictions | **Examples**  What do we need to do to fix this toy?  How do you think the child is feeling?  What did you do with daddy yesterday?  What else did you do at the zoo?  **Non-example**  What else?  And then what? |

**Coding Process**

1. Open up the excel file copy of the SALT transcript you will be coding.
2. **Level 1 coding:** During the first pass through the transcript indicate if each utterance not spoken by the target child (C), is child directed (CDS) towards the target child. Possible speakers include mothers (M), fathers (F) and siblings (S, B).
   1. At this stage, each utterance spoken by parents or siblings will be coded as one of three options: child-directed speech (CDs), other directed speech (OCD) or unclear (UN)
   2. Once all transcripts have been coded at the child-directed level, agreements/disagreements will be calculated with second coder and consensus will made on all disagreements.
3. **Level 2 coding:** During the second pass through the transcript, for each utterance that was coded as child-directed, you will determine the nature of OTR (i.e., expressive [EX] or receptive [RE])
4. **Level 3 coding:** During the third pass and final pass through you will indicate if the expressive OTR was explicit (OTR-E) or implicit (OTR-I), the type of ORT (e.g., yes/no question, comment etc.) and code the complexity of explicit OTRs (e.g., Management). Note that at this level you are only handling expressive OTRs
   1. Refer to Tables 1 & 2 for specific codes.
5. Once you have completed step 2 (level 1 codes) or have completed the full coding procedure save the coded file as a new file, adding your initials and level of coding complete, (e.g., LENA SALT P04_ME_L1, LENA SALT P04_ME_L12) and mark it in the project tracking sheet.

**Tips and Tricks**

**Level 1: Child Directed Speech (CDS) vs Other-directed speech (ODS) vs Unclear (UN)**

Deciding if an utterance is CDS or ODS will be relatively straightforward during dyadic interactions (target child and one communicative partner), as it can be assumed that all utterances are directed at the child, however there may still be instances where an ODS or UN code would be appropriate. In triadic, quadratic or quintet interactions, it may be harder to determine if the utterance is CDS or not. In these instances, use contextual cues to make a decision. For example:

- If the proceeding utterances are between two communicative partners other than the target child code the utterances as ODS. In the following example, all four utterances would be coded as ODS.
  - F: What is your favorite sport? [ODS]
  - S: soccer [ODS]
  - F: Do you want to watch the game together? [ODS]
  - S: Yes [ODS]
- If its clear from proceeding utterances that the target child and a communicative partner are engaging in a certain activity (reading a book, coloring etc) and the utterance is not related to the activity, code as ODS or unclear. In the example below, we can assume the father and child are engaged in a coloring activity, the first utterance is not related to the activity so can be coded as ODS. In the second utterance is unclear if it is CDS or ODS so is coded as UN.
  - F: What show is playing right now? [ODS]
  - F: Look at that! [UN]
  - C: XX
  - F: what color are you going to make the flower? [CDS]
- You’ll need intelligible utterances to determine if an utterance is CDS or ODS. For example, in the following situation, the utterances will be marked as unclear in LENA transcripts.
  - C: look I found this
  - S: XX [UN]
  - C: I like this
  - S: XX [UN]

**Level 2**

Deciding on Expressive vs Receptive OTR.

- Is the utterance intended to evoke an expressive response or a behavioral comply/directive that is NOT phrased in a yes/no format? For example: Put your coat on (RE) vs Can you put your coat on (EX). Even though the parent may intend for a yes/no question phrase to be directive, by framing it as a question it provides an opportunity for the child to expressively respond.
- If an utterance just includes a child’s name, code as EX, as a typical response may be gaze shift and verbal acknowledgement e.g., yes, uhuh

**Level 3**

Type of Expressive OTRs

- For open-ended questions: Even if a question has a correct response e.g. what does a cat say, the format in which the child can respond is open-ended e.g. they can say meow, woof, no, I don’t know etc. and therefore this is coded as open-ended.

Complexity of Explicit OTRs

- Management OTRs are often used to maintain conversations e.g., what else, and then what?
- If the utterance is requesting information that would be perceptually available to the child e.g., it is clear they are reading a book and the caregiver says “what is that?” or what do you see? it would be coded as LLC. If after that to keep the conversation going they say “what else” what would be coded as maintain, as the intent is to keep the conversation going.
