# Supplementary figures and images for: The complexity of opportunities to respond used by mothers and fathers of children with Down syndrome: A preliminary investigation
Source: J Child Lang. Author manuscript; Available in PMC 2025 Aug 2. (PMC11946924; doi:10.1017/S0305000924000370)

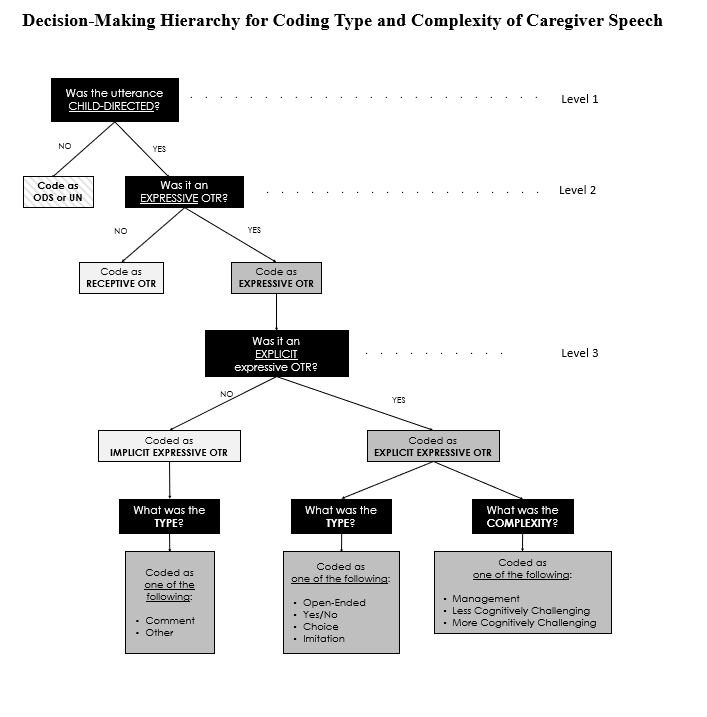

Supplement: Supplementary Material 3 [file NIHMS2041977-supplement-Supplementary_Material_3.docx]
